# A high-performance deep reservoir computer experimentally demonstrated with ion-gating reservoirs

**DOI:** 10.1038/s44172-024-00227-y

**Published:** 2024-06-19

**Authors:** Daiki Nishioka, Takashi Tsuchiya, Masataka Imura, Yasuo Koide, Tohru Higuchi, Kazuya Terabe

**Affiliations:** 1 Research Center for Materials Nanoarchitectonics (MANA), National Institute for Materials Science (NIMS), 1-1 Namiki, Tsukuba, Ibaraki 305-0044 Japan; 2 Department of Applied Physics, Faculty of Science, Tokyo University of Science, Katsushika, Tokyo 125-8585 Japan; 3 Research Center for Functional Materials, NIMS, 1-1 Namiki, Tsukuba, Ibaraki 305-0044 Japan; 4 Research Network and Facility Services Division, NIMS, 1-2-1 Sengen, Tsukuba, Ibaraki 305-0047 Japan

**Keywords:** Electronic devices, Electronic properties and materials

## Abstract

While physical reservoir computing is a promising way to achieve low power consumption neuromorphic computing, its computational performance is still insufficient at a practical level. One promising approach to improving its performance is deep reservoir computing, in which the component reservoirs are multi-layered. However, all of the deep-reservoir schemes reported so far have been effective only for simulation reservoirs and limited physical reservoirs, and there have been no reports of nanodevice implementations. Here, as an ionics-based neuromorphic nanodevice implementation of deep-reservoir computing, we report a demonstration of deep physical reservoir computing with maximum of four layers using an ion gating reservoir, which is a small and high-performance physical reservoir. While the previously reported deep-reservoir scheme did not improve the performance of the ion gating reservoir, our deep-ion gating reservoir achieved a normalized mean squared error of 9.08 × 10^−3^ on a second-order nonlinear autoregressive moving average task, which is the best performance of any physical reservoir so far reported in this task. More importantly, the device outperformed full simulation reservoir computing. The dramatic performance improvement of the ion gating reservoir with our deep-reservoir computing architecture paves the way for high-performance, large-scale, physical neural network devices.

## Abstract (web summary)

Daiki Nishioka and colleagues show a nanodevice implementation of deep reservoir computing using an ion-gating reservoir, achieving record-low error rates on a complex computational task. This device is more efficient and scalable for brain-like computing systems exploiting physical systems.

## Introduction

Physical reservoir computing (PRC), which directly utilizes the nonlinear dynamics inherent in physical systems for information processing, has attracted attention in recent years because it can drastically reduce the computational resources required for information processing^1,2^. Nonlinearity, short-term memory, and high dimensionality are required for reservoirs that map input data nonlinearly into a high-dimensional feature space^3,4^, and physical devices with these features are promising for PRC^1^. Many types of physical reservoirs have been reported, including memristors, optical devices, spintronics devices, soft bodies, nanowire networks, and ion-gating reservoirs. Further, information processing, including image recognition, spoken digit recognition, and time series prediction tasks, have been demonstrated using such physical reservoir devices^1,2,5–27^. The efficiency of PRC based on material-based computation makes it particularly promising for application to resource-limited edge AI devices. However, the computational performance of PRC is still insufficient for practical information processing tasks performed by such material-based efficient edge AI devices (e.g., dynamic image recognition and time series analysis tasks for in-situ processing of time series output from sensors, such as blood glucose level prediction and speech sentence recognition), an approach is required in order to substantially improve performance. One way to achieve this is deep reservoir computing (deep-RC), in which reservoirs are multilayered. This method is expected to be a promising approach, just as neural networks (NN) have been shown to achieve high expressive power and performance by utilizing deep layering. In full-simulation reservoir computing (RC), multilayering of the reservoir parts has been considered, and it has been reported that layering small reservoirs can improve performance in comparison to single-layered reservoirs with the same total reservoir size^28–33^. Furthermore, it has been reported that not only deepening the reservoir layer but also training the connection weights between layers according to the task improves the expressiveness of the network and the flexibility of the model, as well as the computational performance and efficiency^29,30^. In particular, Deep-Echo State Network, with hyperbolic tangents as a nonlinear function, has shown remarkable performance improvements in the prediction tasks of nonlinear autoregressive moving average models and chaotic dynamical systems when the connection weights between the layers are trained by linear regression with the targets^29,30^. On the other hand, few attempts at multilayering in physical reservoirs or physical NN have been reported, and those that have been reported are limited to methods that either do not train the connection weights between reservoirs (i.e., the network is not highly flexible)^34,35^ or train the connection weights between reservoirs (or layers of physical NN) using backpropagation algorithms that require complex calculations that rely on external circuits and have large computational costs^33,36^. It is particularly notable that there are no reports of deep-RC with nanodevices that are advantageous for integration to realize practical AI devices, and thus, it is not clear that multilayering is effective in improving the performance of physical reservoirs.

Here, as the first implementation of nanodevice-based Deep-RC, we describe a demonstration of deep physical reservoir computing using an ion-gating reservoir (IGR), which is a compact and high-performance physical reservoir. The IGR is a nanodevice with a transistor structure consisting of a hydrogen-terminated diamond channel and a Li^+^ electrolyte (Li-Si-Zr-O)^23,37^, and the ion-electron coupled dynamics based on the electric double-layer (EDL) effect in the nanoregion near the Li^+^ electrolyte/diamond interface is used as nonlinear dynamics for the reservoir computing.

There are two approaches to achieving high-performance PRC: one is to improve the performance by modifying the physical reservoir itself, and the other is to maximize the information processing capability of the physical system through system approaches such as optimization of the network structure and external data manipulation (e.g., pre-processing such as masking). In this study, we employed the latter approach, which improves PRC performance through deep network architecture, and used the IGR, which has achieved high computational performance in time series data analysis and image recognition tasks^23,26^, as a model device for this approach.

First, we verified the computational performance of the deep-RC scheme reported for simulation-RC using IGR^28–30^. In this network, the connection weights between reservoir layers are trained based on a simple linear regression algorithm, which provides a higher network flexibility compared to the scheme in which the connection weights between reservoirs are not trained^34,35^, and does not require a back-propagation algorithm. The backpropagation algorithm is an effective method that greatly improves the expressive power of the network, but it is difficult to apply to PRCs based on complex and dynamic nonlinearities (black box functions) originating from physical systems because it requires detailed information on the nonlinearities in the reservoir layer and their derivatives^33,36^. In this respect, the method of learning weights between layers by linear regression using targets is well suited for implementation in physical systems because it does not require detailed information on nonlinearities in physical systems (and their derivatives, etc.) and does not require inverse input of errors to physical systems in order to backpropagate the errors^28–30^. However, the conventional scheme with a simple series structure network reported for the simulation-RC does not improve the computational performance of the IGR. This was found to be due to the inherent characteristic of general physical reservoirs, which are sensitive to input conditions. In addition, this scheme does not provide any improvement in network size limitation, which is one of the main reasons that severely inhibited the performance of conventional PRCs. Whereas in simulation-RC it is easy to increase the reservoir size to the desired performance in exchange for computational cost, in PRCs the number of reservoir states (current and voltage response, mechanical vibration, optical response, etc.) obtained from the physical device is limited by the means of access to the physical system, such as measurement probes. Thus, it is difficult to increase the network size of such PRCs.

On the other hand, a deep-RC scheme with parallel structure overcome such limitations of physical reservoirs in principle, and succeeded in greatly increasing the number of reservoir states by utilizing the reservoir outputs of the previous layer as well as the final outputs among the layered device outputs. As a result, the performance of the IGR applied with the subject deep-RC scheme (deep-IGR) was drastically improved, with a 41% reduction in error compared to single-layer IGR in a second-order nonlinear autoregressive moving average (NARMA2) task. However, the model also showed an increase of unnecessary reservoir states leading to overlearning. Therefore, a modified model was developed to improve this model by evaluating the high dimensionality of the reservoir state in terms of the correlation coefficient between reservoir states, the number of layers was increased by excluding featureless reservoir states (which do not contribute to high dimensionality), which resulted in a 53% reduction in error for the NARMA2 task compared to single-layer IGR, with a normalized mean squared error (NMSE) of 9.08 × 10^−3^. This is the best performance of any physical reservoir reported to date^22–25,38–40^, outperforming a full-simulation RC^38^ for the NARMA2 task.

## Results

### Ion-gating reservoirs using electric double-layer transistors

**Fig. 1:**
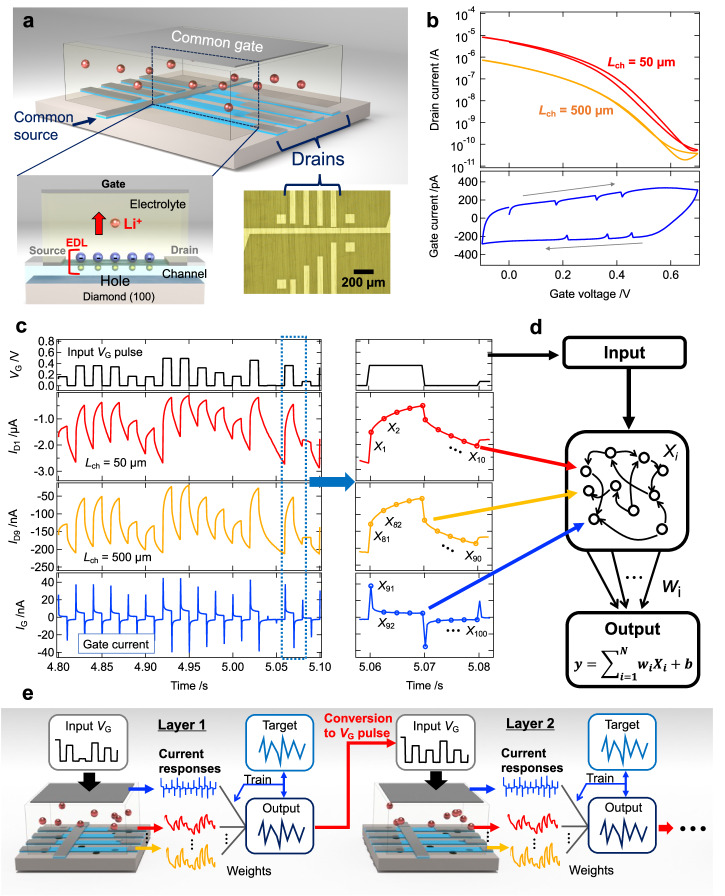
Physical reservoir computing using an ion gating reservoir (IGR). **a** Schematic of the IGR transistor. The inset is an optical microscope image of a diamond with source and drain electrodes. **b** *I*_D_-*V*_G_ curves (upper panel) for channels with lengths of 50 µm and 500 µm and *I*_G_-*V*_G_ curve (lower panel). **c** *I*_D_ responses (*L*_ch_ = 50 µm, 500 µm) and *I*_G_ response to pulsed *V*_G_ input. The right-hand panel shows how to obtain virtual nodes from such current responses. **d** Schematic of reservoir computing. **e** Schematic of the deep reservoir computing implemented by IGRs.

To demonstrate the applicability of the deep-RC architecture, we employed an IGR as the physical reservoir part, implemented with an EDL transistor composed of a Li^+^ electrolyte (Li-Si-Zr-O) and hydrogen-terminated diamond, as shown in Fig. 1a. The IGR transistor has 9 channels of different lengths *L*_ch_ (=5, 10, 25, 35, 50, 100, 250, 350, 500 µm), and the 9 drain current responses can be nonlinearly transformed by the EDL mechanism to the input gate voltage signal. Figure 1b shows the drain current (*I*_D_)-gate voltage (*V*_G_) curves obtained from channels with lengths of *L*_ch_ = 50 µm and *L*_ch_ = 500 µm (upper panel) and a gate current (*I*_G_) – *V*_G_ curve (lower panel). With a positive gate voltage applied, Li^+^ in the electrolyte accumulates at the electrolyte/diamond interface, and electrons are injected into the diamond, which is a hole conductor, resulting in a high resistance state of the diamond. On the other hand, when a negative gate voltage is applied, negatively charged Li vacancies in the electrolyte accumulate on the diamond surface, forming an EDL at the electrolyte/diamond interface as shown in Fig. 1a. Then, holes are doped into the hydrogen-terminated diamond channel, resulting in a low-resistivity state and the drain current is nonlinearly modulated^37^. As different channel resistances exhibit different relaxation times for different channel lengths, if the gate input is a pulse signal, it will exhibit different drain current responses depending on the channel length, as shown in Fig. 1c, providing a higher dimension as a physical node^23^. The structure of this device, where different channel lengths coexist, provides current responses with different relaxation times. The coexistence in one system of a current response with a short relaxation time, which reflects a short-term experience and has strong nonlinearity, and a current response with a long relaxation time, which reflects a long-term experience and has relatively weak nonlinearity, can be expected to provide high performance^1,23,41^. In addition to these drain currents, the gate currents, obtained from the input gate terminals, show a spiked response, as shown in the bottom panel of Fig. 1c, which provides additional diversity to the IGR^24,26^. In redox-based IGR, the use of gate currents as reservoir states has been reported to improve computational performance^24^. In addition to the 10 physical nodes obtained by adding the gate current response to the 9 drain current responses, 10 nodes per pulse response were obtained as virtual nodes, as shown in the right-hand panel of Fig. 1c. Thus, the number of reservoir states *X*_*i*_ obtained from the IGR (i.e., nodes) is 100. Said reservoir states *X*_*i*_ were normalized from 0 to 1 and subsequently used for the reservoir part in the schematic of reservoir computing shown in Fig. 1d. The reservoir output *y*(*k*) at discrete time step *k* is obtained by the linear combination of reservoir state *X*_*i*_(*k*) and readout weight *W*_*i*_ as follows;

1 $$\begin{array}{c}y\left(k\right)={\sum}_{i=1}^{N}{{W}_{i}X}_{i}\left(k\right)+b\\ ={{{{{\boldsymbol{WX}}}}}}\left(k\right)+b\end{array}$$

where *b* is the bias; *N* is the reservoir size; ***W*** = (*W*_1_, *W*_2_, …, *W*_*N*_) is the readout weight vector; ***X***(*k*)=[*X*_1_(*k*), *X*_2_(*k*), …, *X*_*N*_(*k*)]^T^ is the reservoir state vector. Figure 1e is a schematic of the deep-IGR which is a physically implemented deep reservoir computing with IGRs. In the first layer, as in conventional IGRs^23^, the voltage-transformed input signal was input to the gate of the IGR, and the reservoir output was calculated by a linear sum of the weights and reservoir states (Eq. 1) obtained by acquiring virtual nodes from the obtained current responses, as shown in Fig. 1c. The weights were trained by linear regression so that the target and reservoir outputs matched. In deep-IGR, the reservoir output of the first layer is converted to a voltage pulse and input to the gate of the second layer IGR, and the reservoir output is obtained via weights using the obtained current response as in the first layer. The input that reproduced the target waveform to some degree in the first layer is again nonlinearly transformed by IGR into a higher dimensional feature space for learning, which allows the representation of target features that could not be represented in the first layer. The number of said layers can be increased by inputting the reservoir outputs of the previous layer, in the same way as in the second layer, for the third and subsequent layers. The performance of deep-IGR was evaluated by the error between the target and the reservoir output obtained by the input and forward propagation of a dataset different from the training dataset, with the weights of all layers fixed. The details of deep-IGR are discussed later in this document.

**Fig. 2:**
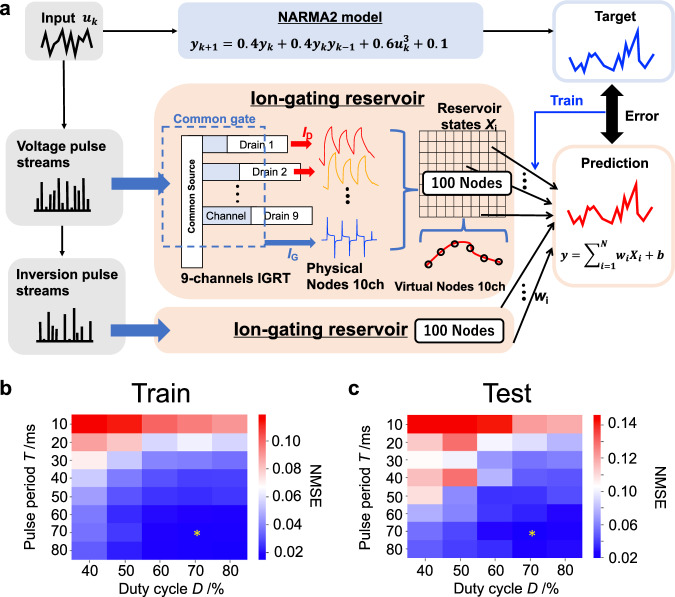
Performance evaluation of a single ion gating reservoir (IGR) by the second-order nonlinear autoregressive moving average (NARMA2) task. **a** Schematic of the NARMA2 task performed by IGR, showing the pulse period *T* and duty cycle *D* dependence of normalized mean squared errors (NMSEs) for training (**b**) and test (**c**) phases. Optimal conditions are indicated by *.

Before evaluating the performance of deep-IGR, we performed a NARMA2 task that predicts the NARMA2 model shown in Eq. 2 following a prior study^42^, in order to evaluate the computational performance of the single-layer IGR.

2 $${y}_{{{{{\rm{t}}}}}}(k+1)=0.4{y}_{{{{{\rm{t}}}}}}(k)+0.4{y}_{{{{{\rm{t}}}}}}(k){y}_{{{{{\rm{t}}}}}}(k-1)+{0.6u}^{3}(k)+0.1$$

where *y*_t_(*k*) are the model outputs; *u*(*k*) are the random inputs, ranging from 0 to 0.5. The NARMA2 task, which is widely used as a benchmark task for physical reservoirs, requires reservoirs to exhibit second-order nonlinearities and short-term memory^22–25,38–40^. Figure 2a shows a schematic of the NARMA2 task performed by IGR. The random input signal *u*(*k*) was converted into voltage pulse streams with an intensity of 0 V to 0.5 V, a pulse period of *T*, and a duty cycle of *D*, then input to the gate terminal of the IGR transistor. The responses of the drain current to the gate voltage pulse streams were measured at a constant drain voltage of −0.5 V. As shown in Fig. 1c, 100 reservoir states *X*_*i*_ were obtained from 10 current responses and virtual nodes (*i* = 1, 2, …, 100). Furthermore, an additional 100 reservoir states *X*_*i*_ (*i* = 101, 102, …, 200) were obtained by applying an intensity-reversed input *u*_inv_(*k*) [= 0.5 - *u*(*k*)] to the IGR (inversion pulse method), resulting in a total of 200 reservoir states, the combination of which was utilized to obtain the reservoir output as shown in Eq. 1. Details on the inversion pulse method and reservoir size of the single IGR are given Supplementary Note 1 and Supplementary Figs. S1-3. In the training phase, the readout weights were trained by linear regression, in order to match the reservoir output *y*(*k*) and the model output *y*_t_(*k*). Details of the training algorithm are given in the Method section herein. In the test phase, performance was evaluated by NMSE (Eq. 3) of the reservoir output (prediction) *y*(*k*) to the model output *y*_t_(*k*) for a different input *u*(*k*) than in the training phase.

3 $${{{{{\rm{NMSE}}}}}}=\frac{1}{M}\frac{{\sum }^{M}_{{k}={1}}{\left[{y}_{{{{{\rm{t}}}}}}\left(k\right)-y\left(k\right)\right]}^{2}} {{\sigma }^{2}\left[{y}_{{{{{\rm{t}}}}}}\left(k\right)\right]}$$

where *M* is the data length (*M* = 1600 for the training phase and *M* = 700 for the test phase); *σ*^2^(·) is the variance. Figure 2b and c show the pulse period *T* and duty cycle *D* dependence of NMSEs in the training and test phases, respectively. The best results, for both training and test phases, were obtained at *T* = 70 ms and *D* = 70%, where the NMSE was 0.0157 in the training phase and 0.0194 in the test phase. Supplementary Fig. S4a shows the *D* dependence of NMSE at *T* = 70 ms, and Supplementary Fig. S4b shows the *T* dependence of NMSE at *D* = 70%. Both of these are minima at the optimal conditions (indicated by *), indicating that the search for optimal conditions in single-layer IGR was successfully performed. In other words, this is the limit of the computational performance that can be achieved by adjusting the pulse period and duty cycle. Further performance improvements require consideration of voltage conditions (*V*_G_ and *V*_D_) and preprocessing of the input signal (feature extraction, masking, etc.). Tuning these so-called ‘hyperparameters’ is difficult for physical reservoirs that require actual measurements, and the huge variety of parameters (voltage, time, temperature, number of masks, etc.) and their combinations make it extremely difficult to maximize the potential computational performance of the physical system. In all of the deep-IGR experiments discussed below, the voltage pulse conditions were fixed at *T* = 70 ms and *D* = 70%. In this case, the optimal conditions match between training and testing, but if such is not the case, the optimal conditions in the training data should be adopted as the input conditions for the second and subsequent layers in order to avoid incorrect optimization by the testing data.

### Performance evaluation of deep ion-gating reservoir with NARMA2 task

**Fig. 3:**
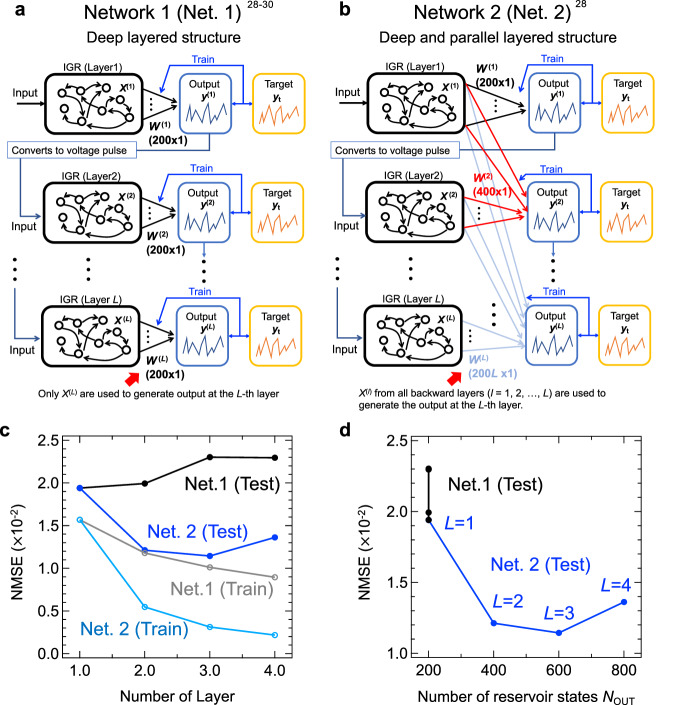
Performance evaluation of a deep-ion gating reservoir (deep-IGR) for Network 1 and Network 2 by the second-order nonlinear autoregressive moving average (NARMA2) task. Schematic diagram of deep-IGR for (**a**) Network 1^28–30^ and (**b**) Network 2^28^. **c** NMSEs of the NARMA2 task vs. the number of layers of deep-IGR. The black and blue plots show the results for Network 1 and Network 2, respectively. **d** Normalized mean squared errors (NMSEs) of the NARMA2 task vs the number of reservoir states used to generate output in each layer.

We experimentally demonstrated the deep-IGR shown in Fig. 3a,b as a framework that overcomes the limitations discussed above, and easily maximizes the computational performance of the physical system. Although the deep layered network structure shown in Fig. 3a (Network 1) has been reported to improve performance in a full simulation reservoir^28–30^, there are no reports of its application to a physical reservoir. In the first layer of Network 1, the readout weight *W*^(1)^ is trained with the same procedure as with the single-layer IGR shown in Fig. 2a, so as to obtain the first-layer reservoir output *y*^(1)^(*k*). In the *L*-th layer$({L}\geqq{2})$, the voltage-transformed reservoir output *y*^(*L*−1)^(*k*) from the previous layer is input to the IGR instead of random input *u*(*k*). The reservoir output of the *L*-th layer is then calculated by the linear combination of the reservoir state *X*^(*L*)^(*k*) obtained from the IGR and the readout weight *W*^(*L*)^ trained by linear regression. In the test phase, the signal was propagated forward with the weights of all layers fixed, and the computational performance was evaluated by the error between the reservoir output y^(*L*)^(*k*) and the model output *y*_t_(*k*) (Eq. 2) at the final layer *L*. For details on the operating time in our Deep-RC scheme, please refer to Supplementary Note 3 and Supplementary Figs. S5 and S6. The black and gray plots in Fig. 3c show the dependence of the NMSE on the number of layers in the test phase and the training phase, respectively, for the NARMA2 task performed on Network 1 to the training data. In this task, the reservoir is used to predict the NARMA2 model output *y*_t_(*k*) from input *u*(*k*). However, in the structure of Network 1 (deep layered), the nature of the problem changes after the second layer, and the task switches to predicting the NARMA2 model output using the reservoir prediction of the previous layer as input (task switching). In a physical reservoir that uses the transient response of a physical system in real time, the properties of the reservoir state (nonlinearity, memory capacity, and diversity) change strongly depending on the operating conditions (in this case, the input *V*_G_ pulse stream conditions), so that the accuracy in a given task changes due to sensitivity to the device operating conditions (i.e., as discussed in Fig. 2, the performance varies greatly with the operating conditions of the IGR.). Therefore, in this case, where the nature of the task has changed, different operating conditions for the IGR need to be explored because different characteristics are required for the reservoir. However, the method of searching for optimal operating conditions for each additional layer is not realistically achievable. In addition, although the task does not change considerably after the second layer, the input to the reservoir (i.e., the output of the previous layer) changes according to the learning status of the network, so optimization of the operating conditions is still considered necessary.

Therefore, in order to overcome such drawback and improve performance, without the need to search for optimal operating conditions, we considered Network 2 (Fig. 3b). This network is a modified model of Network1 (deep layered) that utilizes all the reservoir states obtained from the reservoirs of the previous layers [*X*^(1)^(*k*), *X*^(2)^(*k*),…, *X*^(*L*)^(*k*)] so as to obtain the reservoir output of a given *L*-th layer (deep and parallel structure). In this scheme, higher dimensionality (the number of reservoir states used to obtain output) increases with the number of layers, so that the expressiveness of the reservoir also increases. Another major difference from Network 1 (deep layered) is that task switching does not occur as discussed in Network 1. Since the reservoir state *X*^(1)^(*k*) in the first layer is always used in the computation, regardless of the number of layers, the reservoir states in the second and subsequent layers can be interpreted as additional features that improve the accuracy of predicting the NARMA2 model from the reservoir states in the first layer. The blue and light blue plots show the dependence of the NMSE on the number of layers in the test phase and the training phase, respectively, for the NARMA2 task performed on Network 2 (deep and parallel layered)^28^. The NMSE decreases dramatically as the number of layers increases, and the error decreases by 41% at the third layer compared to the single layer. Figure 3d shows the relationship between NMSEs and the number of reservoir states used to generate output in final layer (*N*_OUT_) for the two networks. Network 2 (deep and parallel layered) clearly shows a reduction in errors compared to Network 1(deep layered). This is possibly due to the fact that the number of nodes was effectively increased, while such increase is generally difficult to achieve with physical reservoirs, as mentioned above. However, in Network 2 (deep and parallel layered), the error increased slightly at the fourth layer, which increase effectively halted the performance improvement.

### Node Selection in deep-IGR

**Fig. 4:**
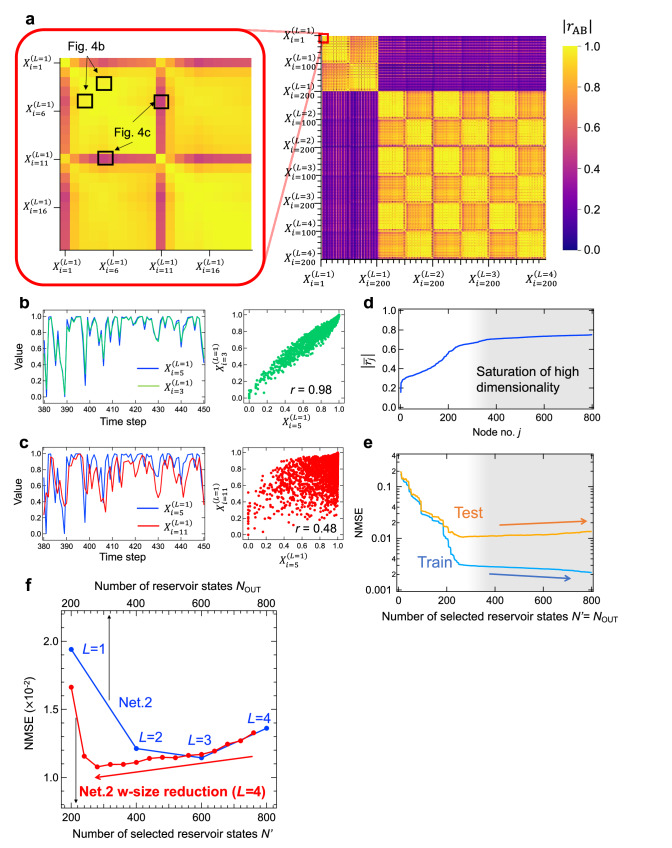
Node selection in deep-ion gating reservoir (deep-IGR). **a** Heatmap of the correlation coefficients of the reservoir states used in the output for the 4th layer of Network 2. An example of (**b**) a strongly correlated or (**c**) relatively weakly correlated reservoir state and a scatterplot. **d** The $\left|\bar{{r}_{j}}\right|$ plots, rearranging the node numbers *j* in order from the lowest $\left|\bar{{r}_{j}}\right|$. **e** The relationship between the number of selected nodes *N*’ and the normalized mean squared error (NMSE) of the second-order nonlinear autoregressive moving average (NARMA2) task. **f** *N*_OUT_ vs NMSEs of NARMA2 task.

The decrease in performance with respect to the increase in the number of nodes, which is shown in Fig. 3d, is thought to originate from the effect of overfitting due to the increase in the number of unnecessary nodes^43^. Overfitting in linear regression is determined by the combination of reservoir size and training data length. Therefore, if the training data length is limited, overfitting will occur as the reservoir size increases (especially the number of unnecessary nodes). To verify this hypothesis, we analyzed the high dimensionality in the correlation coefficient *r*_AB_ between node A and node B shown in Eq. 4 for the reservoir state used at the output of the fourth layer of this Network 2^24^.

4 $${r}_{{{{{{\rm{AB}}}}}}}=\frac{{\sum }_{k=1}^{M}\left({X}_{{{{{{\rm{A}}}}}}}\left(k\right)-{\bar{X}}_{{{{{{\rm{A}}}}}}}\right)\left({X}_{{{{{{\rm{B}}}}}}}\left(k\right)-{\bar{X}}_{{{{{{\rm{B}}}}}}}\right)}{\sqrt{{{\sum }_{k=1}^{M}\left({X}_{{{{{{\rm{A}}}}}}}\left(k\right)-{\bar{X}}_{{{{{{\rm{A}}}}}}}\right)}^{2}{{\sum }_{k=1}^{M}\left({X}_{{{{{{\rm{B}}}}}}}\left(k\right)-{\bar{X}}_{{{{{{\rm{B}}}}}}}\right)}^{2}}}$$

where *X*_A_(*k*) is the reservoir state of node A and ${\bar{X}}_{{{{{{\rm{A}}}}}}}$ is the average value of *X*_A_(*k*). Figure 4a shows the correlation coefficients |*r*_AB_| between all reservoir states ${X}_{i}^{\left(L\right)}$ (*i* = 1 ~ 200, *L* = 1 ~ 4) used to generate output in the fourth layer of Network 2 (i.e., 800 nodes in total). In the first layer, a random wave *u*(*k*) was input to the IGR, whereas in layer *L* (≧2), the output y^(*L*−1)^(*k*) of the previous layer was input to the IGR. Therefore, because these correlation coefficients are small, due to the difference in inputs, the |*r*_AB_| in the first layer is very different from that in the rest of the layers, as shown in Fig. 4a. The inset of Fig. 4a shows an expanded part of the reservoir state in the first layer. The other major correlated/uncorrelated node combinations were also achieved by the following node combinations. (1) gate current/drain current, (2) original pulse/inverted pulse, (3) virtual node corresponding to pulse on time/pulse interval, etc. For example, the correlation coefficient |*r*| between ${X}_{i=5}^{\left(L=1\right)}$ and ${X}_{i=3}^{\left(L=1\right)}$ is close to 1, and they exhibit similar behavior, as shown in Fig. 4b. They correspond to different virtual nodes taken from the same drain current (*L*_ch_ = 5 µm). On the other hand, ${X}_{i=5}^{\left(L=1\right)}$ and ${X}_{i=11}^{\left(L=1\right)}$ have a relatively low correlation coefficient |*r*| of 0.48 and exhibit different behavior, as shown in Fig. 4c. These nodes correspond to different virtual nodes taken from different drain currents (*L*_ch_ = 5 µm and *L*_ch_ = 10 µm).

To identify which of the 800 nodes shown in Fig. 4a has the lower correlation coefficient (i.e., contributes to the higher dimensionality), the average value of the correlation coefficient for node *j* (=1, …, 800) was calculated as shown in Eq. 5;

5 $$\left|\bar{{r}_{j}}\right|=\frac{{\sum }_{i\ne j}^{{N}_{{{{{{\rm{all}}}}}}}}\left|{r}_{{ji}}\right|}{{N}_{{{{{{\rm{all}}}}}}}-1}$$

where *N*_all_ is the number of all nodes (in this case, *N*_all_ = 800). The $|\bar{{r}_{j}}|$ plots, rearranging the node numbers *j* in order from the lowest $|\bar{{r}_{j}}|$, are shown in Fig. 4d. The lowest $|\bar{{r}_{1}}|$ is 0.3, whereas $|\bar{{r}_{j}}|$ increases with increasing *j*, indicating that $|\bar{{r}_{j}}|$ saturates at about *j* = 300. This suggests that about 500 of the total 800 node reservoir states are nodes that do not contribute to high dimensionality (i.e., the nodes are less effective for performing the given task). To evaluate the effect of node correlation coefficients and high dimensionality on computational performance, the NARMA2 task was performed by increasing number of selected reservoir states *N*’ in the order of lower $|\bar{{r}_{j}}|$ (for example, when *N*’ = 100, *X*_*j*_(*j* = 1 ~ 100) was used for the calculation). Note that *N*’ = *N*_OUT_ in this case, where node selection is made only on the final layer (*L* = 4). Figure 4e shows the relationship between *N*’ and NMSE; in the region where *N*‘ is approximately 260 or less, *N*’ increases while NMSE decreases for both training and testing. On the other hand, in the region where *N*‘ is above approximately 260, *N*’ increases and the training error continues to decrease, while the test error increases. Also, as shown in Supplementary Fig. S7, the derivative of the test error converges to a slightly positive value in the region where *N*’ is above about 270. This indicates the effect of overfitting with increasing reservoir size, in which reservoir states in regions of saturated diversity have a negative impact on the computation. Figure 4f shows the results obtained with size reduction (Fig. 4e) and the relationship between the NMSEs of the NARMA2 task (test phase) and *N*_OUT_ for Network 2. The red plots show the results of the computation when gradually excluding nodes, in order of largest $|\bar{{r}_{j}}|$, from the 800 nodes which were used to generate output in the fourth layer of Network 2. It was found that the NMSE decreased despite any reduction in the number of reservoir states used to generate the output. Reducing to *N*’ = 600 (i.e., excluding from the calculation the 200 nodes with large $|\bar{{r}_{j}}|$), the improvement in computational performance was only about the same as in the third layer without size reduction, but at *N*’ = 460 (i.e., excluding from the calculation the 340 nodes with large $|\bar{{r}_{j}}|$), it rather outperformed an interpolated version of Network 2 without size reduction between *L* = 2 and *L* = 3. The performance continued to improve as the number of nodes was reduced to about *N*’ = 300, but when *N*’ was further reduced, the performance rapidly degraded due to the loss of the nodes necessary for the computation being performed. These results indicate that the adverse effects of multilayering, such as unnecessary node growth and overfitting, were successfully suppressed without sacrificing the advantages of improved computational performance due to multilayering. Here, network pruning was performed by selectively incorporating uncorrelated nodes in the network through correlation coefficient analysis. This pruning method effectively exploits the feature of RC that its computational performance is achieved by high dimensionality. Therefore, the pruning method based on the correlation coefficient is useful for identifying nodes that lead to higher dimension and identifying performance improvement mechanisms that lead to higher dimension and performance improvement, although other methods, such as network pruning based on network structure analysis can also be used in our Deep-RC.

### Deep-IGR architecture, which utilizes node selection at each layer

**Fig. 5:**
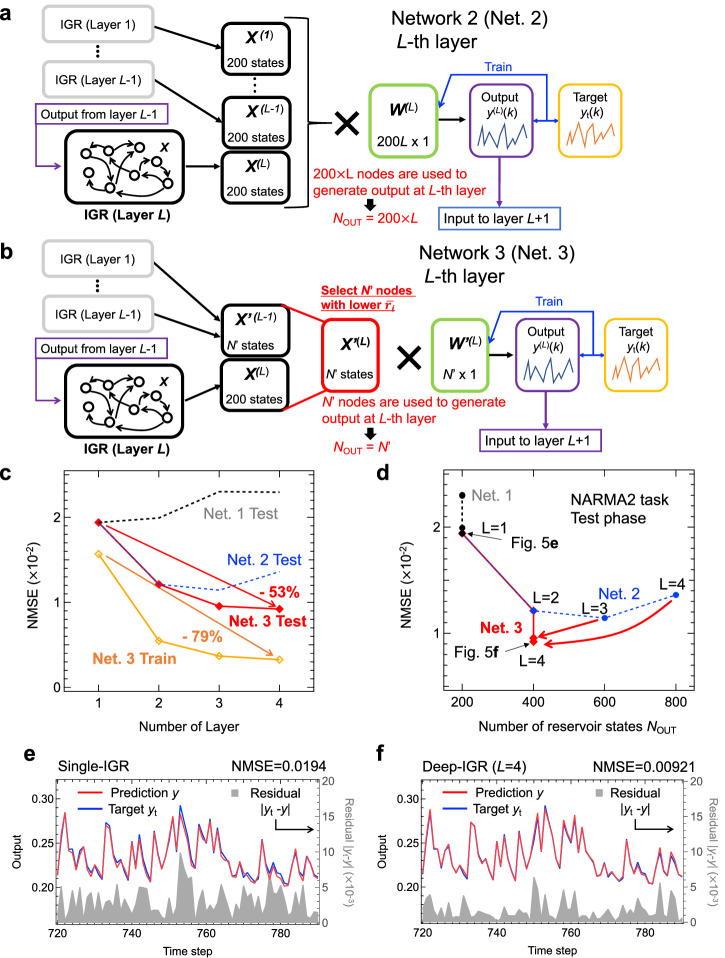
Performance evaluation of a deep-ion gating reservoir (deep-IGR) for Network 3 by the second-order nonlinear autoregressive moving average (NARMA2) task. Schematic diagram of layer *L* of a deep-IGR for (**a**) Network 2 and (**b**) Network 3 with node selection. **c** Normalized mean squared errors (NMSEs) of the NARMA2 task vs. the number of layers of deep-IGR. **d** NMSEs of the NARMA2 task vs *N*_OUT_. Target waveform and predicted waveform by IGR at (**e**) *L* = 1 and (**f**) *L* = 4.

**Fig. 6:**
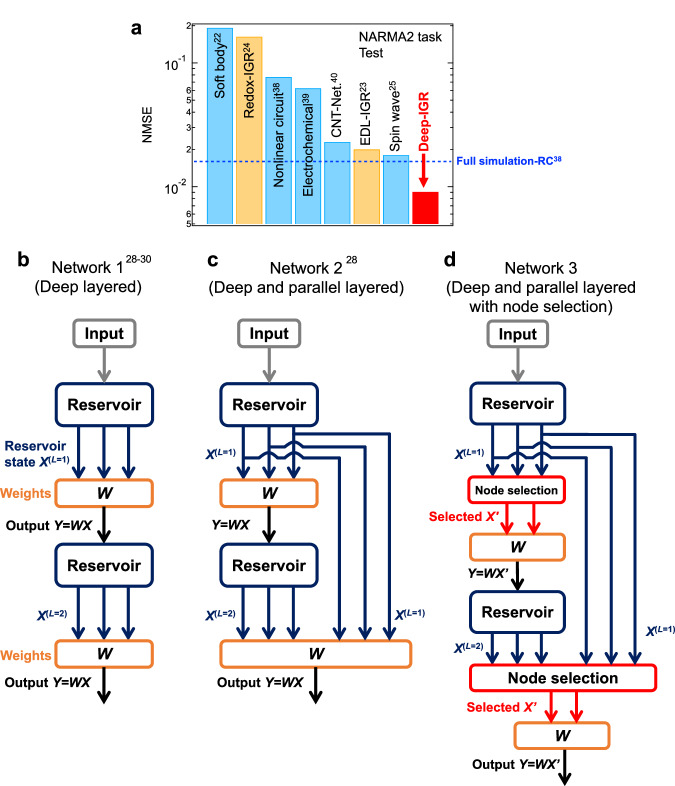
Performance comparison with other physical reservoirs and summary of deep-reservoir computing (deep-RC) structures in this study. **a** Performance comparison with other physical reservoirs by the second-order nonlinear autoregressive moving average (NARMA2) task^22–25,38–40^. The result for soft body^22^ was converted to the normalized mean squared error (NMSE) in Eq. 3 for comparison. Schematic diagram of the Deep-RC architecture for physical reservoir computing (PRC) investigated in this study for (**b**) Network 1^28–30^, (**c**) Network 2^28^ and (**d**) Network 3.

The node selection (reduction) by $|\bar{{r}_{j}}|$ was adapted to only one layer in the configuration shown in Fig. 4. Here, in order to maximize computational performance, we consider Network 3, in which node selection is adapted to all layers from the second layer onward by modifying Network 2 (deep and parallel layered). The number of selected reservoir states *N*’ used for the output of each layer was set to 400. Therefore, the first (single) and second layers, where the number of reservoir states *N*_OUT_ is 200 and 400, respectively, do not perform node selection and are therefore identical to Network 2, represented in Fig. 3b. The calculation method used for the third and subsequent layers of Network 3 is explained below. The output in the third layer of Network 2 used 600 reservoir states of *X*^(*L*=1)^, *X*^(*L*=2)^ and *X*^(*L*=3)^ [here, these reservoir states are rewritten as *X*_*j*_ (*j* = 1, 2,…, 600)]. In Network 3, of these 600 reservoir states, *N*’ nodes (here 400 nodes) selected as *X*’^(*L*=3)^ in order of smallest $|\bar{{r}_{j}}|$ are used to generate the output of the third layer *y*^(*L*=3)^. Furthermore, by voltage converting *y*^(*L*=3)^ and inputting it to IGR again, *X*^(*L*=4)^ (200 nodes) is obtained, and together with the reservoir states *X’*^(*L*=3)^ (400 nodes) obtained in the previous layers, there are a total of 600 reservoir states [here, these reservoir states are rewritten as *X*_*j*_ (*j* = 1, 2,…, 600)]. As above, *N*’ (=400) of these 600 nodes with small $|\bar{{r}_{j}}|$ were selected *X*’^(*L*=4)^ and used to generate output in the fourth layer *y*^(*L*=4)^. Figure 5a and b show schematic diagrams of how the reservoir outputs *y*^(*L*)^ of Network 2 and Network 3 in layer *L* are computed, respectively. In Network 2, shown in Fig. 5a, the number of reservoir states used for output increases by 200 as the number of layers increases, because all reservoir states *X*^(*L*=1)^, *X*^(*L*=2)^, …, *X*^(*L*)^ in layers 1 to *L* are used to generate the reservoir output in layer *L*. On the other hand, in Network 3, shown in Fig. 5b, X’^(*L*)^ selected by *N*’ nodes in order of smallest $|\bar{{r}_{j}}|$ of the reservoir state *X*’^(*L*−1)^ selected up to the previous layer and the reservoir state *X*^(*L*)^ in Layer *L*, as described above. Therefore, the number of reservoir states used to generate output is fixed at *N*’, regardless of the number of layers *L* (>1). Figure 5c shows a plot of NMSE vs. the number of layers in the NARMA2 task for Network 3 (deep and parallel layered with node selection), with the number of selected nodes *N*’ = 400. The NMSE continued to decrease as the number of layers increased, for both training and testing errors, with the fourth layer achieving better computational performance than Network 2 (deep and parallel layered), shown by the dotted blue line, with an NMSE of 0.00326 in the training phase and 0.00921 in the testing phase. These values are 79% and 53% lower in the training and testing phases, respectively, compared to a single-IGR. Figure 5d shows the relationship between the number of nodes used for output *N*_OUT_ and NMSE (test phase). Network 3 showed improved computational performance over the conventional Network 2, despite *N*_OUT_ after the second layer being fixed at 400 ( = *N*‘). This performance improvement is explained by the network being trained by increasing the number of layers while excluding unnecessary nodes that cause overfitting and nodes that do not contribute to diversity, thereby effectively incorporating the features obtained in each layer. The predicted and target waveforms at layers 1 and 4 are shown in Fig. 5e and f, respectively. Although the predicted waveform in the first layer captured the trend of the target waveform, there was a divergence between the predicted waveform and the target waveform in some areas. On the other hand, the predicted waveforms at the fourth layer, shown in Fig. 5f, are in almost perfect agreement. This shows that the deep-IGR based on Network3 is able to successfully solve the NARMA2 model shown in Eq. 2, and that this architecture drastically improved the computing performance of the IGR. Supplementary Fig. S8 shows the *N’* dependence of NMSE during the test phase of the 3-layer Deep-IGR (Network3). For the deep layered scheme, the test error was minimal when *N’* = 400, therefore, *N’* = 400 is set here. Figure 6a shows a comparison of the computational performance evaluated in the NARMA2 task of this study with other physical reservoirs reported so far^22–25,38–40^. Here, in order to maximize the performance of the deep-IGR, the output weights of the final layer (*L* = 4) of Network 3 were trained with ridge regression. Our deep-IGR (Network 3, *L* = 4) achieved NMSE = 9.08×10^−3^ in the test phase for the NARMA2 task (NMSE = 3.37 × 10^−3^ in the training phase), and achieved the highest computational performance of any physical reservoir reported to date for the NARMA2 task^22–25,38–40^, even outperforming the NMSE = 0.016 of fully simulated reservoir computing [Standard Echo State Network (ESN)]^38^. This standard ESN is a single-reservoir with 300 nodes of a hyperbolic tangent as the activation function^38^, and differs from Deep-IGR (400 nodes and four multilayers) in its network architecture and reservoir size. Although they are not a perfect like-for-like comparison, ESN, which is a typical example of a simulation reservoir, has been widely compared in performance with many physical reservoirs^1,38,44–46^. Simulation reservoirs that can adjust the network structure and reservoir size according to the purpose generally perform better than PRCs, and actually no PRCs that outperform the reported standard ESNs have been reported for the NARMA2 task, as shown in Fig. 6a. Therefore, this comparison has a certain value because our Deep-IGR is the first physical system to outperform this reported simulation reservoir in the NARMA2 task^22–25,38–40^. In particular, the NARMA2 task is one of the best tasks for evaluating and comparing the basic information processing capabilities of PRC, since the task of analyzing second-order dynamic systems is widely performed by PRC^1,10,13,22–25,38–40,47,48^. On the other hand, the NARMA10 task is considered as a more challenging benchmark task, but Deep-IGR was unable to solve the NARMA10 task. This is due to the relatively small memory capacity (MC) of IGR (MC ~ 4). It is expected that if our Deep-RC architecture is implemented in a dynamical system with high memory capacity (e.g., optical elements and spin-wave interference)^5,6,8,25^, the NARMA10 task can be solved with high accuracy. We would like to demonstrate this as future work.

The deep-RC architecture proposed in this study has very few hyperparameters, and can easily enhance the computational performance of physical reservoirs. Usually, it is extremely difficult to maximize the information processing capability of a physical system in physical reservoir computing. There is a wide variety of parameters to be considered if the best computational performance is to be obtained from a physical reservoir; these include the delay time of the feedback loop, the mask matrix used for input signal preprocessing (mask format, number of masks, mask length, etc.), in addition to the intensity and frequency of the signal input to the physical system. Such complexity makes it almost impossible to experimentally search for the best combination of driving conditions for a physical system. Hence, the deep-layered physical reservoir architecture proposed in this study can dramatically improve the computational performance, and can be realized in a small parameter space that is possible to realistically explore. We experimentally examined three different deep reservoir architectures, which are shown in Fig. 6b-d, and found that Network 1 (deep layered, shown in Fig. 6b), which was previously reported for simulated reservoirs, does not necessarily contribute to improved performance in physical reservoirs^28–30^. Said scheme is suitable only as a component part of physical reservoirs, whose characteristics are not so sensitive to operating conditions. Further, it does not increase the network size, which is a serious problem faced by physical reservoirs. On the other hand, we have succeeded in increasing the network size and in improving the computational performance of the physical reservoir drastically with Network 3 (deep and parallel layered with node selection), which is a modified version of Network 2 (deep and parallel layered, shown in Fig. 6c) that selectively uses useful nodes for information processing, as shown in Fig. 6d. These deep-RC schemes should be practically implemented by integrating an FPGA and IGRs, as shown in Supplementary Fig. S9, which we would like to address as future work. Our deep-IGR is the first nanodevice implementation of deep-RC, and the dramatic improvement of the performance of IGR by our architecture provides the possibility of realizing large-scale, brain-like physical devices.

## Conclusion

In this study, we demonstrated the implementation of Deep-RC by nanodevices and the improvement of computational performance achieved by optimizing the deep network structure, whereas Deep-RC has only been implemented by simulated RCs and limited physical reservoirs. We implemented IGR that uses ion-electron coupling dynamics as a computational resource^23^ in the reservoir part, so as to create a multi-layered framework, and found that a simple serial network structure did not improve the performance (Network 1). On the other hand, we determined that a network structure that uses the reservoir state of the previous layer for outputs shows a remarkable improvement in performance (Network 2). This is because, in addition to succeeding in further increasing the dimension, which is generally difficult in physical systems, the predicted output of the reservoir is again input to the IGR and transferred to the high-dimensional feature space, where it is learned so that the deviation from the target output is reduced. In order to suppress overlearning due to increase of unnecessary nodes, which was confirmed in this network structure, node selection based on correlation coefficients was used in Network 3 to effectively extract nodes that are effective for information processing. As a result, an NMSE of 9.08 × 10^−3^ was achieved in the NARMA2 task for deep-IGR. This is the best performance of all the physical reservoirs reported so far for the NARMA2 prediction task which is a typical benchmark task for RCs and PRCs, and even outperforms full-simulation RC^22–25,38–40^. The easy and dramatic performance improvement of IGRs with our deep-RC architecture, and opens the way to the implementation of practical, large-scale, brain-based physical devices.

## Method

### Device fabrication and electrical measurements

Hydrogen-terminated diamond homoepitaxial film was deposited, as a channel, on a IIa-type high-pressure high-temperature single-crystal diamond substrate (100) (EDP) by the microwave-plasma chemical vapor deposition (MPCVD) method. During the deposition process, 500 and 0.5 standard cubic centimeters per minute of H_2_ and CH_4_, respectively, were supplied, and the hydrogen-terminated diamond was grown at a radio frequency of 950 W. The IGR were fabricated with nine different channel lengths (5, 10, 25, 35, 50, 100, 250, 350, and 500 μm), all with a channel width of 50 μm. Pd/Pt electrodes (10 and 35 nm, respectively) were deposited as source and drain electrodes by electron beam evaporation with maskless lithography after oxygen termination of the diamond surfaces (other than channels) by oxygen plasma asher. A 3.5-μm LSZO thin film, used as an electrolyte, was deposited by pulsed laser deposition (PLD) with an ArF excimer laser. A 100-nm Au thin film was deposited by electron beam deposition as a gate electrode.

Electrical measurements of IGR were performed by the source measure unit and pulse measure unit of a semiconductor parameter analyzer (4200A-SCS, Keithley), which measurements were carried out at room temperature inside a vacuum chamber that had been evacuated by a turbo molecular pump. Probers were used to connect the IGR inside the chamber.

### Linear regression algorithm for learning readout networks

In the NARMA2 task, which was used in this study to evaluate performance, the readout network was trained with linear regression using the algorithm described below. The reservoir output shown in Eq. 1 can also be expressed as;

6 $$y\left(k\right)={{{{{\boldsymbol{W}}}}}}\cdot {{{{{\boldsymbol{x}}}}}}\left(k\right)$$

where ***W*** = (*b*, *W*_1_, *W*_2_, …, *W*_*N*_) and ***x***(*k*) = [1, *X*_1_(*k*), *X*_2_(*k*), …, *X*_*N*_(*k*)]^T^ are the weight vector and the reservoir state vector, respectively. The reservoir output **Y** for all training periods (*k* = 1, 2, …, *M*) is described by;

7 $${{{{{\bf{Y}}}}}}={{{{{\bf{WX}}}}}}$$

where **X** = (***x***(1), ***x***(2), …, ***x***(*M*)) and *M* = 1600 are the reservoir state matrix and the data length for the training phase, respectively. The weight matrix that minimizes the squared error is given by;

8 $${{{{{\bf{W}}}}}}={{{{{{\bf{Y}}}}}}}_{{{{{{\rm{t}}}}}}}{{{{{{\bf{X}}}}}}}^{{{\dagger}} }$$

where ${{{{{{\bf{X}}}}}}}^{{{\dagger}} }=[{{{{{{\bf{X}}}}}}}^{{{{{{\rm{T}}}}}}}{\left({{{{{\bf{X}}}}}}{{{{{{\bf{X}}}}}}}^{{{{{{\rm{T}}}}}}}\right)}^{-1}]$ and **Y**_t_ are the Moore-Penrose pseudo-inverse matrix and the target matrix, respectively. In the results shown in Fig. 6a, the readout weights of the final layer of Deep-IGR (Network 3) were trained with the ridge regression as follows.

9 $${{{{{\bf{W}}}}}}={{{{{{\bf{Y}}}}}}}_{{{{{{\rm{t}}}}}}}{{{{{{\bf{X}}}}}}}^{{{{{{\rm{T}}}}}}}{\left({{{{{{\bf{XX}}}}}}}^{{{{{{\rm{T}}}}}}}+\lambda {{{{{\bf{I}}}}}}\right)}^{-1}$$

where *λ* (=2 × 10^−3^) and ${{{{{\bf{I}}}}}}\,(\subseteq {{\mathbb{R}}}^{N\times N})$ are the ridge parameters and identity matrix, respectively. The learning and the sum-of-product calculation performed in the readout network were done on a personal computer using current data obtained from the IGR. However, by utilizing artificial synaptic devices that reproduce weights by conductance, the sum-of-product calculation performed in the readout can also be calculated in a physical process, which is expected to further improve efficiency^49–58^.

### Supplementary information

Supplementary Information

## Data Availability

The datasets generated during and/or analyzed during the current study are available from the corresponding author on reasonable request.

## Code availability

The codes used in the current study are available from the corresponding author on reasonable request.
