# Supplementary material for: A high-performance deep reservoir computer experimentally demonstrated with ion-gating reservoirs
Source: Commun Eng. 2024 Jun 19;3:81. doi: 10.1038/s44172-024-00227-y (PMC11187105; doi:10.1038/s44172-024-00227-y)
Supplement: Supplementary file 1 — Supplementary Information [file 44172_2024_227_MOESM1_ESM.pdf]

## Supplementary information

# A high-performance deep reservoir computer experimentally demonstrated with ion-gating reservoirs


Daiki Nishioka[1,2], Takashi Tsuchiya[1]*, Masataka Imura[3], Yasuo Koide[4], Tohru Higuchi[2], and Kazuya Terabe[1]

[1]Research Center for Materials Nanoarchitectonics (MANA), National Institute for Materials Science (NIMS), 1-1 Namiki, Tsukuba, Ibaraki, 305-0044, Japan.

[2]Department of Applied Physics, Faculty of Science, Tokyo University of Science, Katsushika, Tokyo 125-8585, Japan

[3]Research Center for Functional Materials, NIMS, 1-1 Namiki, Tsukuba, Ibaraki, 305-0044, Japan.

[4]Research Network and Facility Services Division, NIMS, 1-2-1 Sengen, Tsukuba, Ibaraki, 305-0047, Japan.

*Email: TSUCHIYA.Takashi@nims.go.jp

**Supplementary Note 1: Reservoir size and inversion pulse method for the IGR**

This section describes the reservoir size and the method of obtaining the reservoir state and the inversion pulse method for IGRs in the NARMA2 task discussed in Fig. 2. The inversion pulse method is a technique to obtain additional reservoir states by applying to the device inversion pulse streams that is a voltage conversion of the input $u'(k)$, which is the inverted intensity of the original input $u(k)$ shown in Eq. S1.

$$u'(k) = u_{\mathrm{Max}} - u(k) \qquad (S1)$$

, where $k$, $u(k)$, and $u_{\mathrm{Max}}$ are the discrete time, the input, and the maximum value of the input ($u_{\mathrm{Max}}$ = 0.5 for the NARMA2 task), respectively. Supplementary Fig. S1 shows examples of the original input $u(k)$ and the inverted input $u'(k)$. Although they are simply linear transformations, due to the nonlinearity of the mapping function of the reservoir, the reservoir states from both input show completely different behaviors.

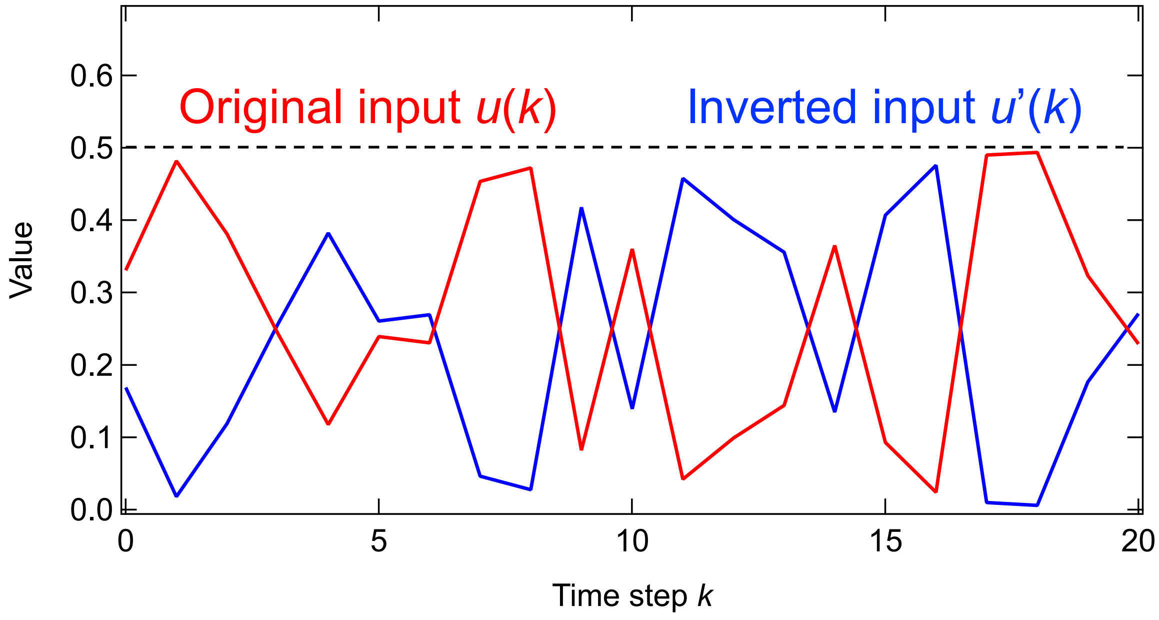


**Supplementary Fig. S1. Original input *u*(*k*) and Inverted input *u*'(*k*) generated by Eq. S1.**

By inputting the original and inverted pulses to the device as shown in Supplementary Fig. S2, a total of 200 (i.e., 10 physical nodes × 10 virtual nodes × 2) reservoir states can be obtained from the IGR. Supplementary Figures S3**a** and **b** shows the current response of the IGR to the original pulse input and the inversion pulse input, respectively. Current responses of the IGR to the inversion pulse shows a completely different behavior, although the inversion pulse is simply a linear transformation of the original input. Supplementary Figures S3**c** and **d** shows an example of reservoir state obtained by the virtual node method for the original and inversion pulse inputs. Ten virtual nodes were obtained per current response by capturing five virtual nodes from the pulse-on sections and five virtual nodes from the pulse-interval intervals. Note that in PRCs that utilize transient responses of physical systems, it is common to use pulsed inputs, and this is equivalent to some kind of masking technique with a 2-

valued binary mask matrix $\boldsymbol{M}$ = (1,0). For the Deep-IGRs discussed in Figure 2 and beyond, 200 reservoir states were generated for each input because of the introduction of such an inversion pulse method in each IGR layer.

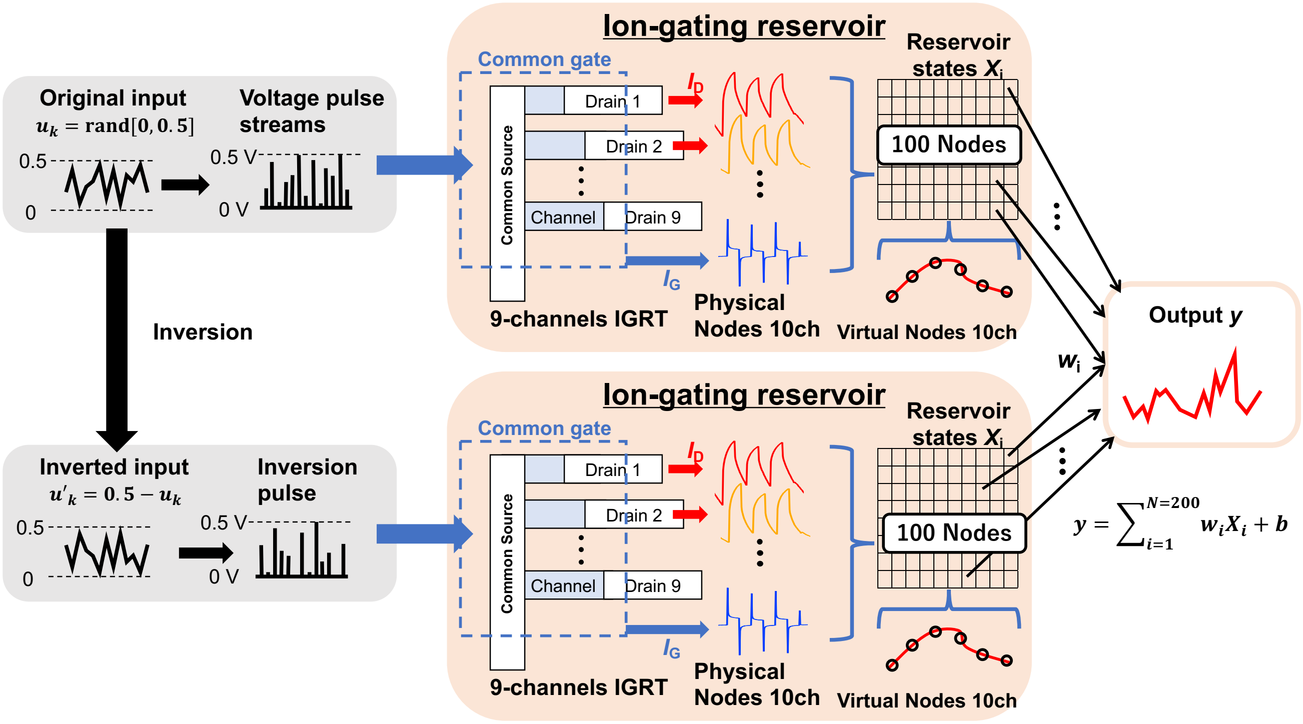


**Supplementary Fig. S2. Schematic diagram of the method for obtaining the reservoir states in IGR using the inversion pulse method.**

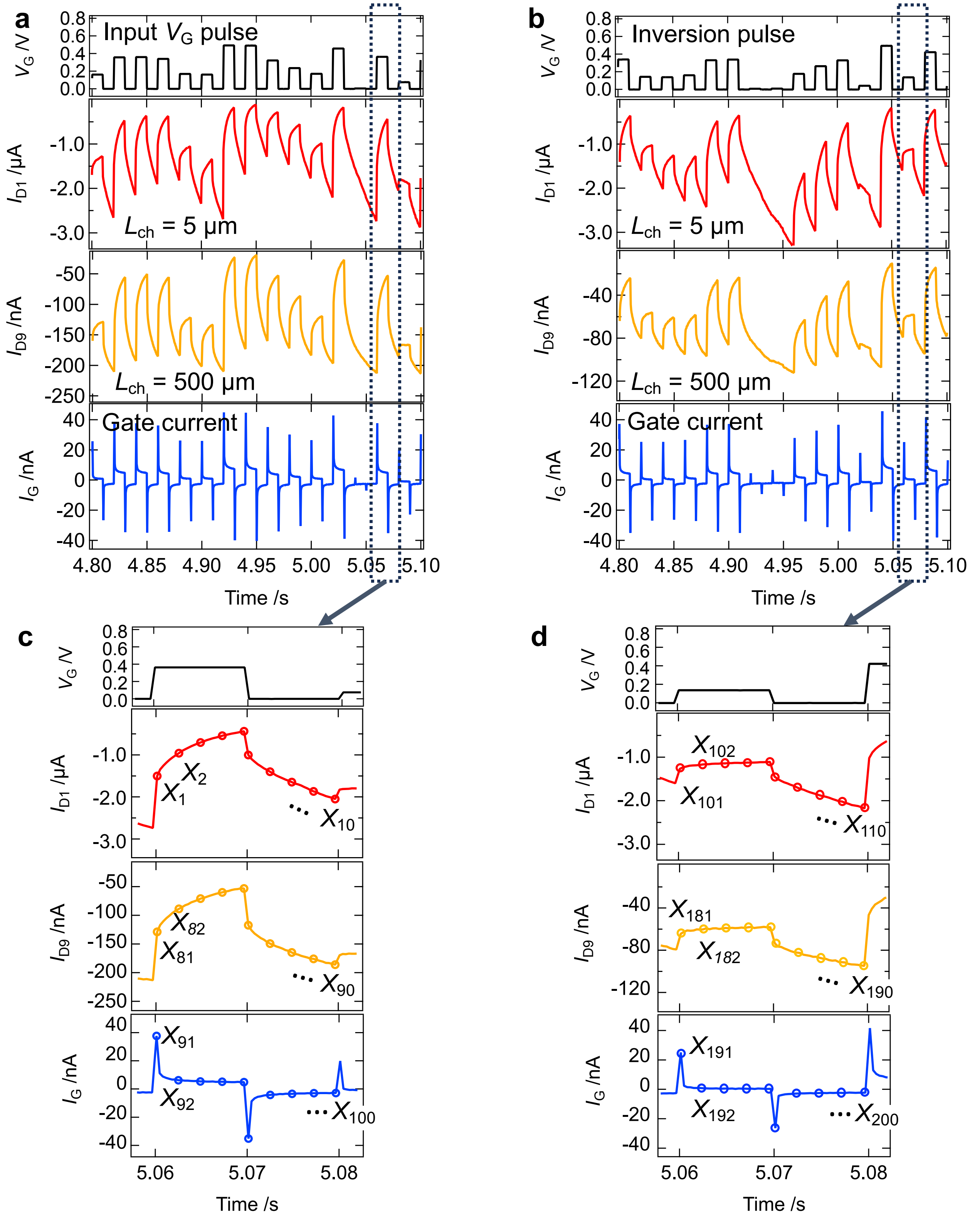


**Supplementary Fig. S3. Reservoir states of IGR using the inverted pulse method.** Drain and gate current responses to (**a**) original pulse input and (**b**) inversion pulse input. Reservoir states of the IGR for (**c**) the original ($X_1$~$X_{100}$) and (**d**) inversion pulse inputs ($X_{101}$~$X_{200}$) obtained by the virtual node method.

## Supplementary Note 2: Pulse period and duty cycle dependence of NMSEs for NARMA2 task in single IGR

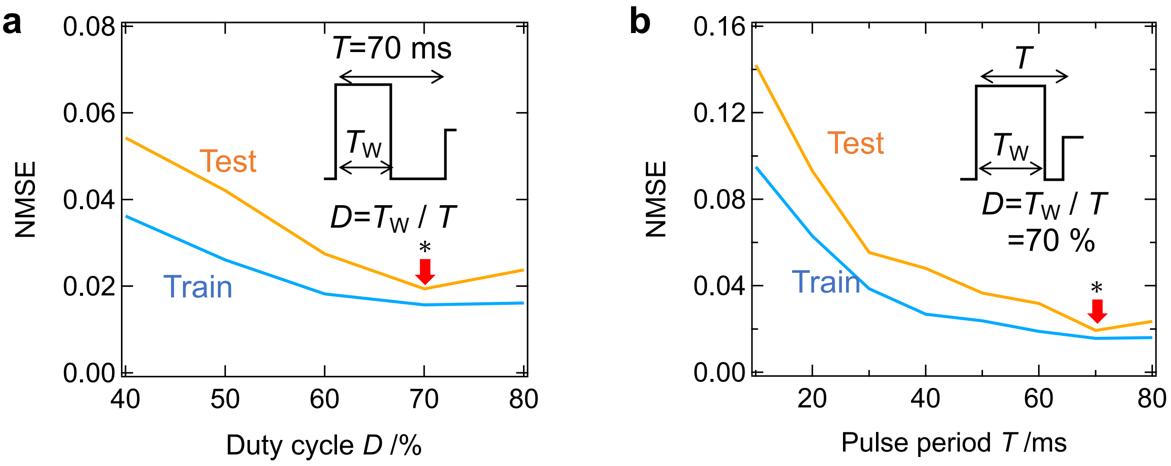


**Supplementary Fig. S4. Pulse period and duty cycle dependence of NMSEs for NARMA2 task.** **a** $D$-dependence of NMSEs at $T$=70 ms and (**b**) $T$-dependence of NMSEs at $D$=70% for NARMA2 task in single IGR. Optimal conditions are indicated by *.

## Supplementary Note 3: Operating time of the Deep-RC scheme

In this section, we discuss the operating time of Deep-IGR. The NARMA2 task described in Fig. 3 was discussed based on a discrete time $k$. However, on the real time scale where IGR actually operates, it takes a time of pulse period $T$ × data length $M$ per layer. Supplementary Figure S5(a) shows a schematic of the Deep-RC scheme's operation during the training phase and its operation time. For each layer $L$, it takes time to generate input pulses (including inverted pulses), to input them to the PRC device (IGR), to train the readout weights $W^{(L)}$, and to calculate the output $Y^{(L)}$, and each operation is performed step by step. Therefore, the $L_{\mathrm{Max}}$-layer Deep-RC scheme requires an operating time $T_{\mathrm{train}}$ as shown in Eq. S2.

$$T_{\mathrm{train}} = \sum_{L=1}^{L_{\mathrm{Max}}} \left[ t_{\mathrm{pulse,gen}}^{(L)} \times M_{\mathrm{train}} + T^{(L)} \times M_{\mathrm{train}} + t_{\mathrm{train}}^{(L)} + t_{\mathrm{output}}^{(L)} \times M_{\mathrm{train}} \right] \tag{S2}$$

, where $M_{\mathrm{train}}$ is the training data length; $t_{\mathrm{pulse,gen}}$ is the time to generate input pulses, $t_{\mathrm{train}}$ is the training time for the weights; $t_{\mathrm{output}}$ is the time to compute the linear sum for output generation. Supplementary Figure S5(b) shows a schematic diagram of Deep-RC operation in the test phase. In the test phase, the operating time $T_{\mathrm{test}}$ of Deep-RC is described by Eq. S3, which excludes the training time of the readout weights from Eq. S2.

$$T_{\mathrm{test}} = \sum_{L=1}^{L_{\mathrm{Max}}} \left[ t_{\mathrm{pulse,gen}}^{(L)} \times M_{\mathrm{test}} + T^{(L)} \times M_{\mathrm{test}} + t_{\mathrm{output}}^{(L)} \times M_{\mathrm{test}} \right] \tag{S3}$$

, where $M_{\mathrm{test}}$ is the test data length. In the case of IGR, $T >> (t_{\mathrm{pulse,gen}}, t_{\mathrm{output}})$ since $T$=70 ms, which is relatively slow. Therefore, the most of the operating time is the device operating time ($T \times M$). As shown in Supplementary Fig. S6, instead of inputting all data $M_{\mathrm{test}}$ to each layer step by step, the operating time can be significantly reduced by adopting a method in which outputs at each discrete time are propagated sequentially to the next layer. In this method, once one discrete-time output is obtained from a layer, it is input to the next layer, and the time $t_{\mathrm{delay}}$ required for this is expressed by the following equation.

$$t_{\mathrm{delay}}^{(L)} = t_{\mathrm{pulse,gen}}^{(L)} + T^{(L)} + t_{\mathrm{output}}^{(L)} \tag{S4}$$

Therefore, the Deep-RC scheme for the $L_{\mathrm{Max}}$ layer requires the operating time $T'_{\mathrm{test}}$ shown in Eq. S5.

$$T'_{\mathrm{test}} = T \times M_{\mathrm{test}} + \sum_{L=1}^{L_{\mathrm{Max}}} t_{\mathrm{delay}}^{(L)} \tag{S5}$$

Since $T \times M_{\mathrm{test}} >> t_{\mathrm{delay}}$ in the case of IGR, the Deep-RC calculation can be performed in almost one layer's operating time, thus the scheme shown in Supplementary Fig. S6 is suitable for the application.

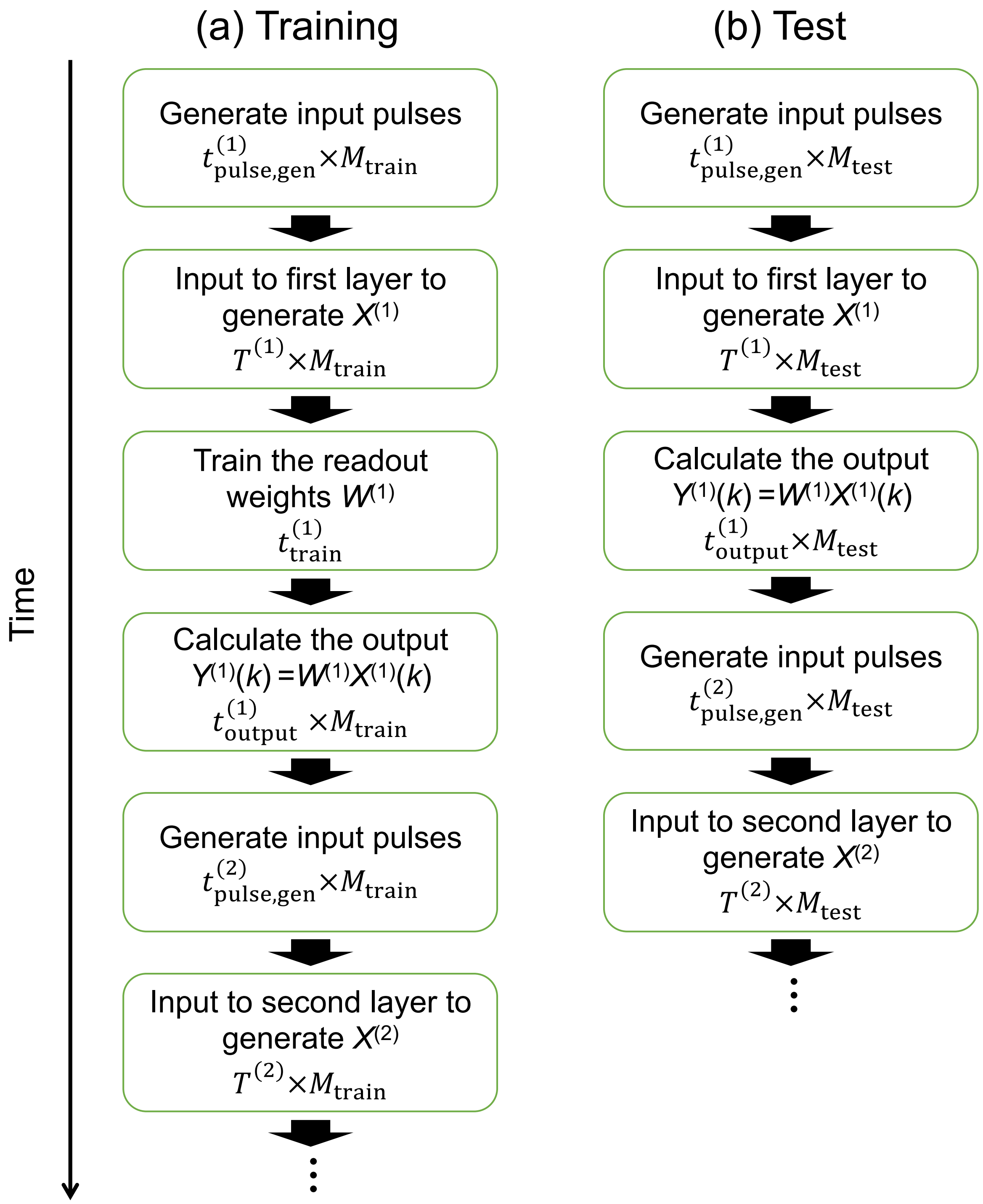


**Supplementary Fig. S5. Operating time of the Deep-RC scheme.** A schematic of the Deep-RC scheme's operation and its operation time for (a) training and (b) test phase.

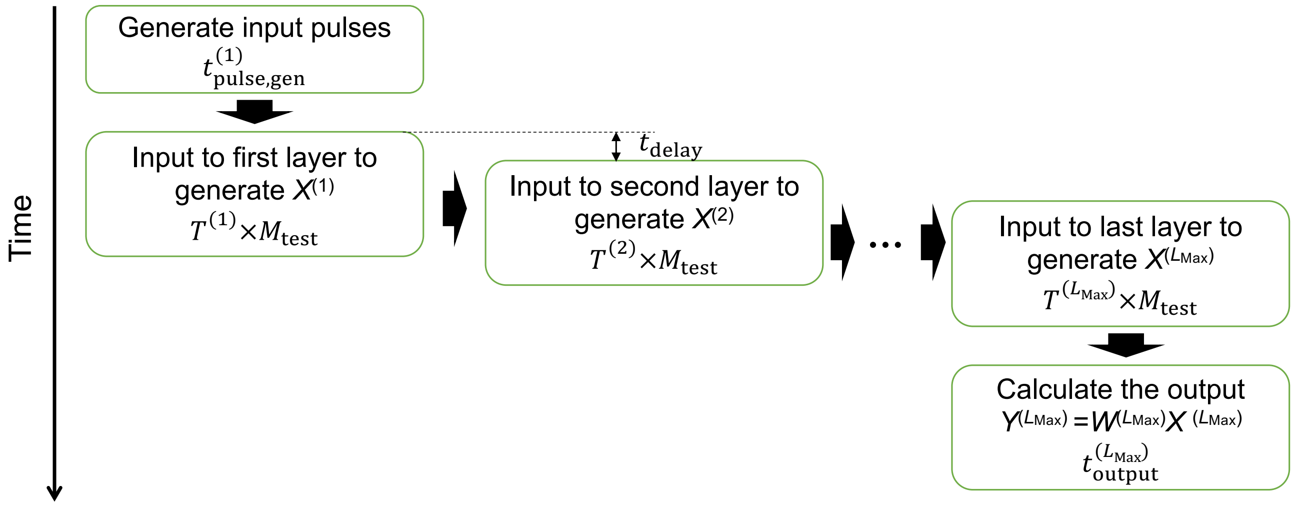


**Supplementary Fig. S6. A schematic of the Deep-RC scheme's operation and its operation time for test phase.**

**<u>Supplementary Note 4: The derivative of the test error with the number of selected nodes in the fourth layer of network 2</u>**

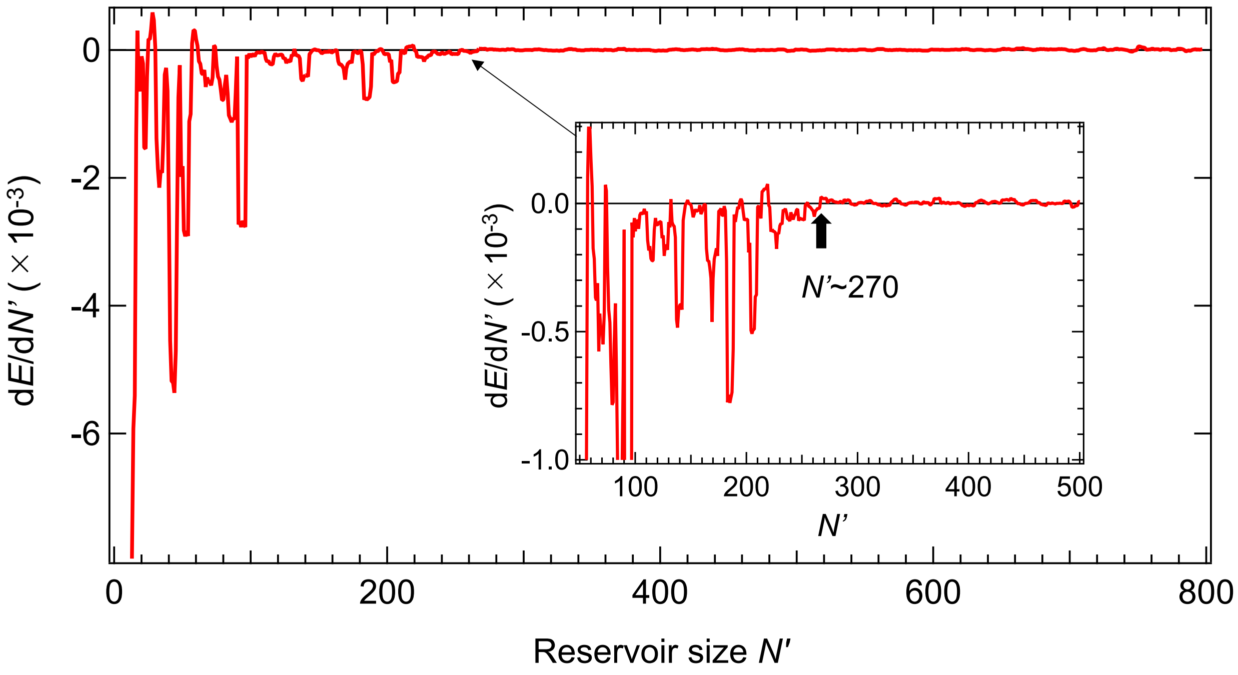


**Supplementary Fig. S7. The derivative of the test error with the number of selected nodes in the fourth layer of Network 2.** *E* represents the test error (NMSE) for the NARMA2 task.

**Supplementary Note 5: $N'$ dependence of NMSE during the test phase of the Deep-IGR for Network3**

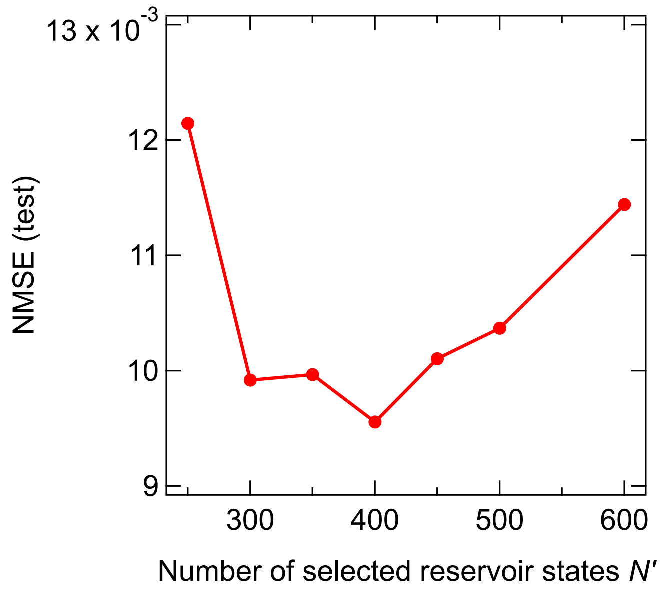


**Supplementary Fig. S8. $N'$ dependence of NMSE during the test phase of the 3-layer Deep-IGR for Network3.** NMSEs are test errors for the NARMA2 task at the third layer of Network 3.

**Supplementary Note 6: Practical architecture for the Deep-IGR**

Here we describe the overall system configuration for implementing the Deep-IGR architecture. Supplementary Figure S9**a** shows a schematic diagram of the system for a single-layer IGR. In order to incorporate the IGR as an actual information processing device, it is necessary to connect the IGR with a computer that performs signal pre-processing, post-processing, and neural network (NN) computations on the readout. For overall system efficiency, these computations should preferably be implemented by an FPGA[1-4)].

First, input information is fed into the FPGA, and pre-processing of the signals, such as D/A conversion and generation of pulse voltage signals, is performed. Next, the generated pulse signal is input to the IGR, and the current response to it is again input to the FPGA to perform digital conversion and obtain the reservoir state. Then, after storing the reservoir state matrix $X$ of the entire waveform in dedicated memory, the output $Y=WX$ is calculated by the digital NN circuit. Among the above processes, most of the processing time is high-dimensional mapping of the input by the IGR, because the processing by FPGA is much faster than the operating speed of IGR (10~100 Hz). Our Deep-IGR architecture can be implemented in the system configuration shown in Supplementary Fig. S9**b**, which is a slight modification of the single-layer IGR system shown in Supplementary Fig. S9**a**. As in the single-layer architecture, the input information is input to the first-layer IGR through the FPGA. The response current is again input to the FPGA, and the output of the first layer $Y^{(L=1)}$ is calculated through digital NN. This output is again converted to analog pulse streams through preprocessing and input to the IGR corresponding to the second layer. The output of the second layer $Y^{(L=2)}$ is then calculated by the FPGA using the same procedure as for the first layer. This architecture can be implemented by performing the above steps up to the maximum number of layers $L_{\mathrm{Max}}$ and outputting the final calculation result $Y^{(L=\mathrm{LMax})}$. Note that the time required for the above operation is shown in Eq. S5 and Supplementary Fig. S6, and most of the operation time is spent on the IGR operating time. In other words, it is not so important to speed up or optimize the processing performed in the FPGA, although optimization of the dedicated circuits in the FPGA may be necessary if a physical system with a fast operating speed is incorporated into the system. In addition, since analog computing in the readout layer using memristor array circuits and optical circuits has been reported in recent years[5-7)], it is expected that more efficient Deep-RC systems can be realized by adopting these approaches.

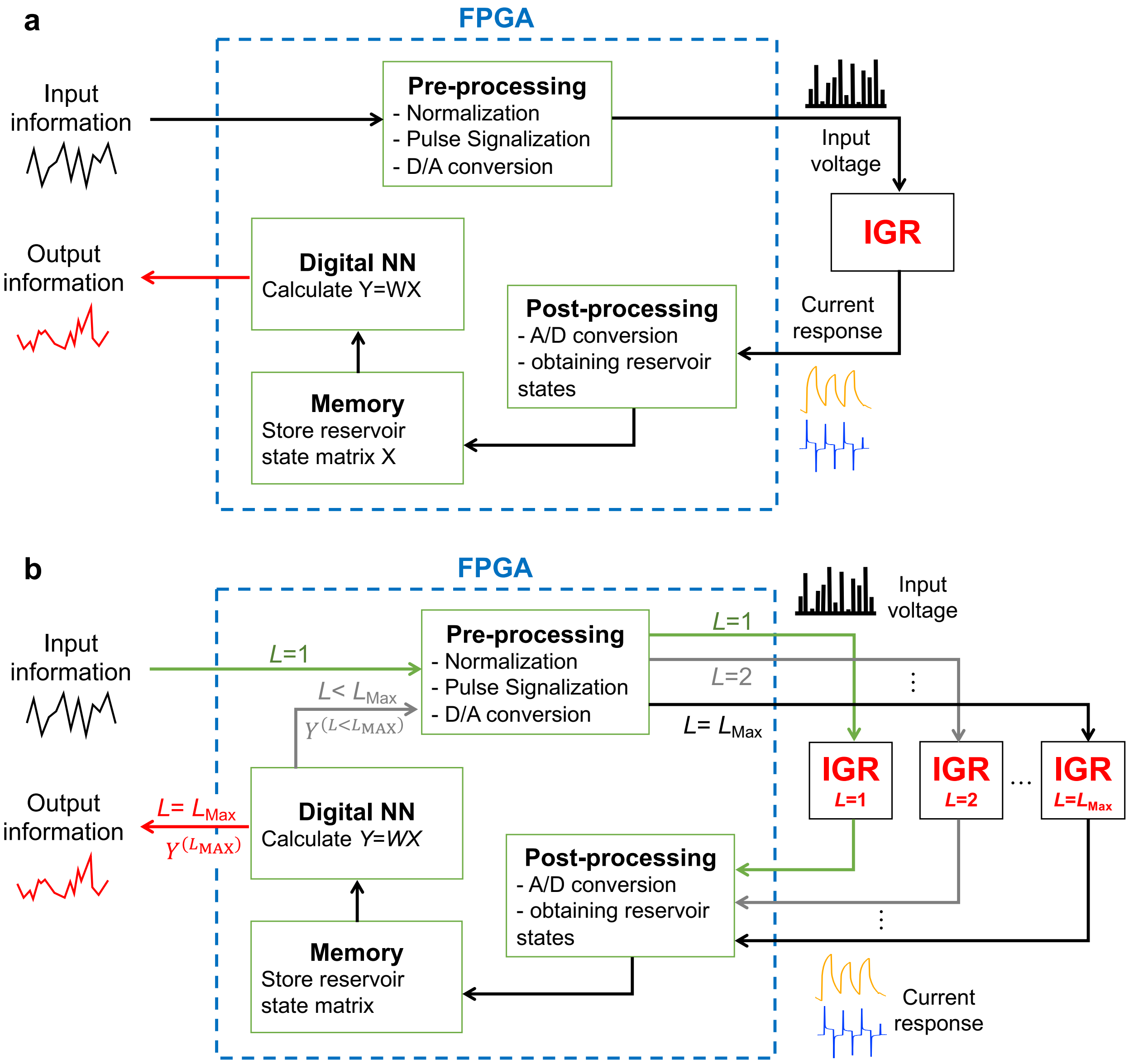


**Supplementary Fig. S9. Schematic diagram of the system for the deep-IGR. a** Single-layer IGR and **b** deep-IGR.

**Supplementary References**


1) Liang, X., Zhong, Y., Tang, J., Liu, Z., Yao, P., Sun, K., Zhang, Q., Gao, B., Heidari, H., Qian, H. & Wu, H. Rotating neurons for all-analog implementation of cyclic reservoir computing. *Nat. Commun.* **13**, 1549 (2022).
2) Sayyaparaju, S., Shawkat, M. S. A., Adnan, M. M., & Rose, G. S. (2020, October). Circuit techniques for efficient implementation of memristor based reservoir computing. *In 2020 IEEE International Symposium on Circuits and Systems (ISCAS) (pp. 1-5). IEEE.*
3) Alomar, M. L., Soriano, M. C., Escalona-Morán, M., Canals, V., Fischer, I., Mirasso C. R. & Rosselló, J. L. Digital Implementation of a Single Dynamical Node Reservoir Computer. *IEEE Transactions on Circuits and Systems II: Express Briefs* **62**, 977-981 (2015).
4) Nakajima, M., Inoue, K., Tanaka, K., Kuniyoshi, Y., Hashimoto, T., & Nakajima, K. Physical deep learning with biologically inspired training method: gradient-free approach for physical hardware. *Nat. Commun.* **13**, 7847 (2022).
5) Zhong, Y., Tang, J., Li, X., Liang, X., Liu, Z., Li, Y., Xi, Y., Yao, P., Hao, Z., Gao, B., Qian, H. & Wu, H. A memristor-based analogue reservoir computing system for real-time and power-efficient signal processing. *Nat. Electron.* **5**, 672-681 (2022).
6) Duport, F., Smerieri, A., Akrout, A., Haelterman, M., & Massar, S. Fully analogue photonic reservoir computer. *Sci. Rep.* **6**, 22381 (2016).
7) Lupo, A., Picco, E., Zajnulina, M., & Massar, S. Deep photonic reservoir computer based on frequency multiplexing with fully analog connection between layers. *Optica* **10**, 1478-1485 (2023).
